# Chaotic medical image encryption method using attention mechanism fusion ResNet model

**DOI:** 10.3389/fnins.2023.1226154

**Published:** 2023-07-13

**Authors:** Xiaowu Li, Huiling Peng

**Affiliations:** ^1^Information Department, The Second Affiliated Hospital of Shantou University Medical College, Shantou, Guangdong, China; ^2^School of Computer and Information Engineering, Luoyang Institute of Science and Technology, Luoyang, Henan, China

**Keywords:** artificial intelligence, medical image encryption, deep learning, ResNet, chaotic system, attention mechanism, medical image security frontiers

## Abstract

**Introduction:**

With the rapid advancement of artificial intelligence (AI) technology, the protection of patient medical image privacy and security has become a critical concern in current research on image privacy protection. However, traditional methods for encrypting medical images have faced criticism due to their limited flexibility and inadequate security. To overcome these limitations, this study proposes a novel chaotic medical image encryption method, called AT-ResNet-CM, which incorporates the attention mechanism fused with the ResNet model.

**Methods:**

The proposed method utilizes the ResNet model as the underlying network for constructing the encryption and decryption framework. The ResNet's residual structure and jump connections are employed to effectively extract profound information from medical images and expedite the model's convergence. To enhance security, the output of the ResNet model is encrypted using a logistic chaotic system, introducing randomness and complexity to the encryption process. Additionally, an attention mechanism is introduced to enhance the model's response to the region of interest within the medical image, thereby strengthening the security of the encrypted network.

**Results:**

Experimental simulations and analyses were conducted to evaluate the performance of the proposed approach. The results demonstrate that the proposed method outperforms alternative models in terms of encryption effectiveness, as indicated by a horizontal correlation coefficient of 0.0021 and information entropy of 0.9887. Furthermore, the incorporation of the attention mechanism significantly improves the encryption performance, reducing the horizontal correlation coefficient to 0.0010 and increasing the information entropy to 0.9965. These findings validate the efficacy of the proposed method for medical image encryption tasks, as it offers enhanced security and flexibility compared to existing approaches.

**Discussion:**

In conclusion, the AT-ResNet-CM method presents a promising solution to address the limitations of traditional encryption techniques in protecting patient medical images. By leveraging the attention mechanism fused with the ResNet model, the method achieves improved security and flexibility. The experimental results substantiate the superiority of the proposed method in terms of encryption effectiveness, horizontal correlation coefficient, and information entropy. The proposed method not only addresses the shortcomings of traditional methods but also provides a more robust and reliable approach for safeguarding patient medical image privacy and security.

## 1. Introduction

With the proliferation and widespread adoption of the Internet, textual, vocal, and visual content have become prevalent carriers of information, facilitating more convenient communication. Among these mediums, images convey a greater wealth of direct and immersive information, making them one of the most commonly utilized methods of communication. However, the transmission of image information raises concerns regarding personal privacy, copyright protection, and other security issues, particularly in the context of medical images, which involve sensitive patient data and vital health information. Consequently, ensuring the security of medical image information is of utmost importance (Kaur et al., 2021).

Medical image encryption technology is specifically designed to obfuscate the original information contained within medical images, thereby safeguarding the security of patient data. While traditional encryption algorithms used for textual information, such as the Data Encryption Standard (DES), Advanced Encryption Standard (AES), and Rivest-Shamir-Adleman (RSA) algorithm, have demonstrated excellent performance, they face unique challenges when applied to image encryption (Ye et al., 2021; Luo et al., 2022). The high inter-pixel correlation and redundancy within images hinder the effectiveness of these methods in the context of medical image encryption (Hua et al., 2021). To address these challenges, researchers and experts have turned to chaotic systems, characterized by sensitivity to initial conditions, unpredictability, and pseudo-randomness, as a viable approach for image encryption (Barik and Changder, 2020; Wang and Chen, 2021). While these methods offer relative simplicity and superior encryption effects, they also entail certain security risks. Furthermore, striking a balance between security performance and encryption efficiency remains an ongoing challenge, necessitating further investigation by the research community.

Given the increasing prevalence of image encryption, the development of efficient image decryption methods becomes equally important. Consequently, numerous scholars have dedicated their efforts to exploring effective techniques for cracking chaotic encryption systems. One conventional approach involves direct inference of the encryption key through plaintext attacks (Zhang et al., 2012). However, this method is intricate and requires acquiring the system key prior to initiating the attack. Another approach involves constructing a key dictionary to search for the encryption key (Zhong et al., 2021; Guan and Chen, 2022); however, this method is time-consuming and inefficient. Therefore, it is imperative to explore more efficient techniques for recovering the original image from ciphertext without compromising the integrity of the encryption system.

Deep learning represents a significant advancement and extension of machine learning, offering enhanced capabilities for feature expression. With the continuous improvement in computational power, deep learning has demonstrated promising outcomes in various domains, including image processing (Lu et al., 2023), image steganalysis (Ge et al., 2021), image style transfer (Kim and Choi, 2022), and image reconstruction (Noda et al., 2023). Deep learning models can construct feature extractors with superior performance by leveraging large-scale datasets. In the context of medical image encryption, deep learning methods can effectively incorporate the characteristics of medical images, resulting in improved encryption and decryption functionalities. Notably, deep neural networks (DNN) are deeper fully connected neural networks with enhanced non-linear expression capabilities (Yang et al., 2020). Convolutional neural networks (CNN), a popular variant of deep learning models, excel in extracting hidden deep information from data and have found successful applications in image encryption (Vidhya et al., 2020; Wang et al., 2022). However, deep CNN models face challenges such as gradient disappearance or explosion, which can hinder the search for a global optimal solution. To address this, residual networks (ResNet) have been proposed as a variant of traditional CNN models (He et al., 2016). ResNet overcomes the limitations of network depth by incorporating residual modules, enabling better solutions for image encryption (Zhu et al., 2022). Despite the success of ResNet in image encryption, its application in medical image encryption remains relatively unexplored. Additionally, the attention mechanism, which assigns varying weights to different features, has shown promising results in image processing tasks (Li et al., 2022). Therefore, integrating the attention mechanism into medical image encryption can enhance the quality of the encrypted images.

Building upon the existing literature, this paper proposes a chaotic medical image encryption method that utilizes an attention mechanism fused with a ResNet model. Compared to other approaches, our proposed method offers higher security, flexibility, and a novel solution for protecting patient privacy and sensitive health information. The key contributions of this paper can be summarized as follows:

1. The proposed method leverages ResNet as the backbone network to construct encryption and decryption networks, facilitating the adaptive extraction of high-dimensional feature expressions from the original medical image. This not only enhances the encryption quality of the model but also addresses the issue of gradient disappearance in deep networks through the use of skip connections, resulting in a more flexible and secure encryption model.
2. The encrypted image is obtained through the XOR operation based on a Logistic chaotic system, which achieves the seamless integration of deep learning and chaotic systems. This integration contributes to improved encryption quality in medical images.
3. The introduction of the attention mechanism assigns higher weights to the region of interest within the medical image. This significantly enhances the encryption performance of the model, leading to better encryption outcomes in medical image encryption tasks.

The remainder of this paper is organized as follows: Section 2 provides a comprehensive review of existing literature related to the utilization of deep learning methods for image encryption. Section 3 presents the methodologies employed in this study, along with the evaluation metrics used. Section 4 presents the experimental details, results, and a comparison of evaluation metrics, including the horizontal correlation coefficient and information entropy. Section 5 discusses the experimental findings and analyzes the limitations of the proposed method. Finally, Section 6 summarizes the contributions made in this paper and outlines potential directions for future research.

## 2. Related research

**Figure 1:**
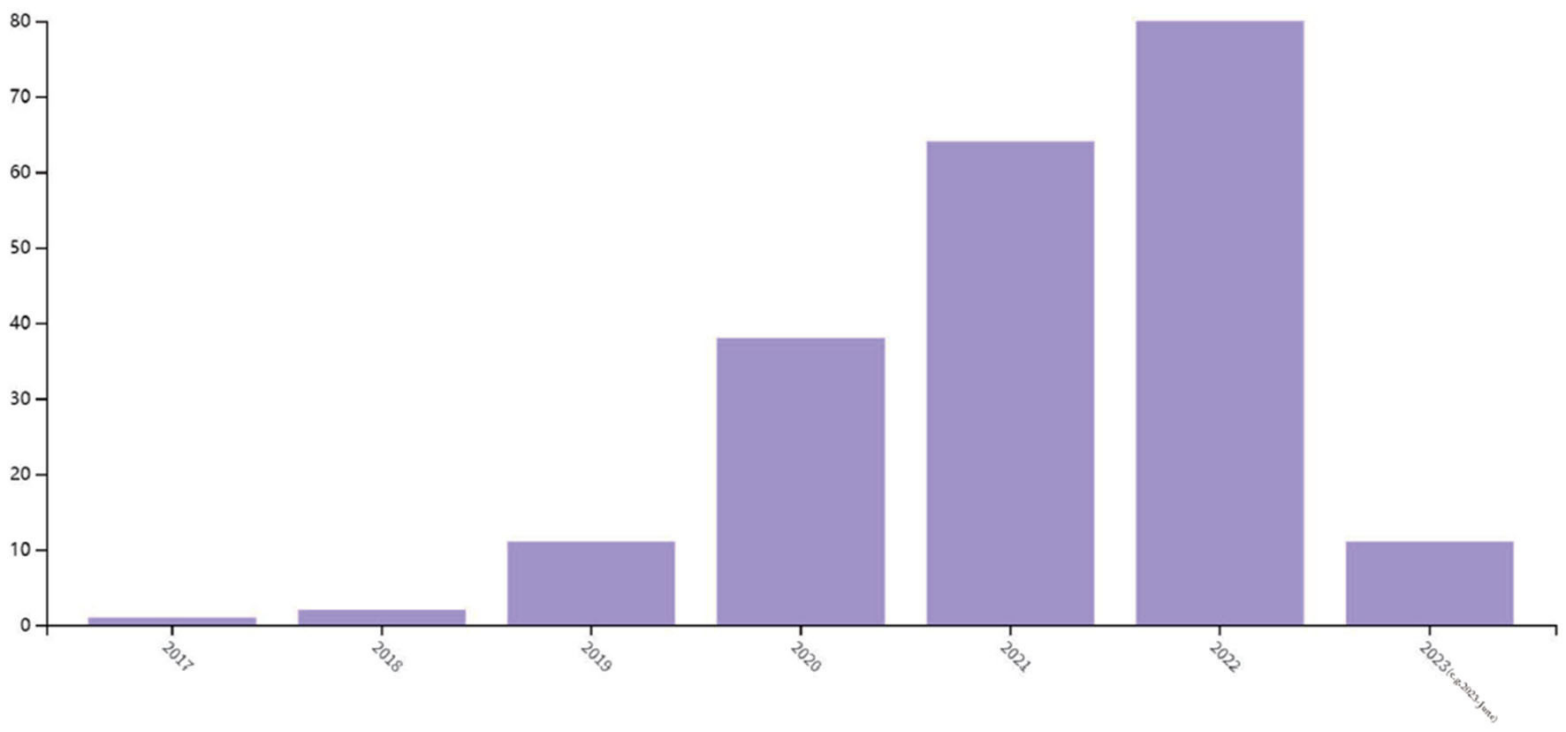
Statistics of image encryption method based on deep learning in 2017–2023 (e.g., 2023-June).

Currently, the integration of deep learning and image encryption is still in its early stages. Figure 1 illustrates the frequency of image encryption methods that employ deep learning in recent years, according to a search conducted on the Web of Science. The graph reveals that the application of deep learning to image encryption tasks has only emerged in recent years, with the number of publications increasing annually. This section aims to review the efforts of various researchers who have attempted to apply diverse deep learning models to image encryption research. The goal is to provide a better understanding of the similarities and differences between deep learning and image encryption systems. Through an analysis of the advantages and limitations of these methods, this paper ultimately introduces the proposed medical image encryption method.

**Figure 2:**
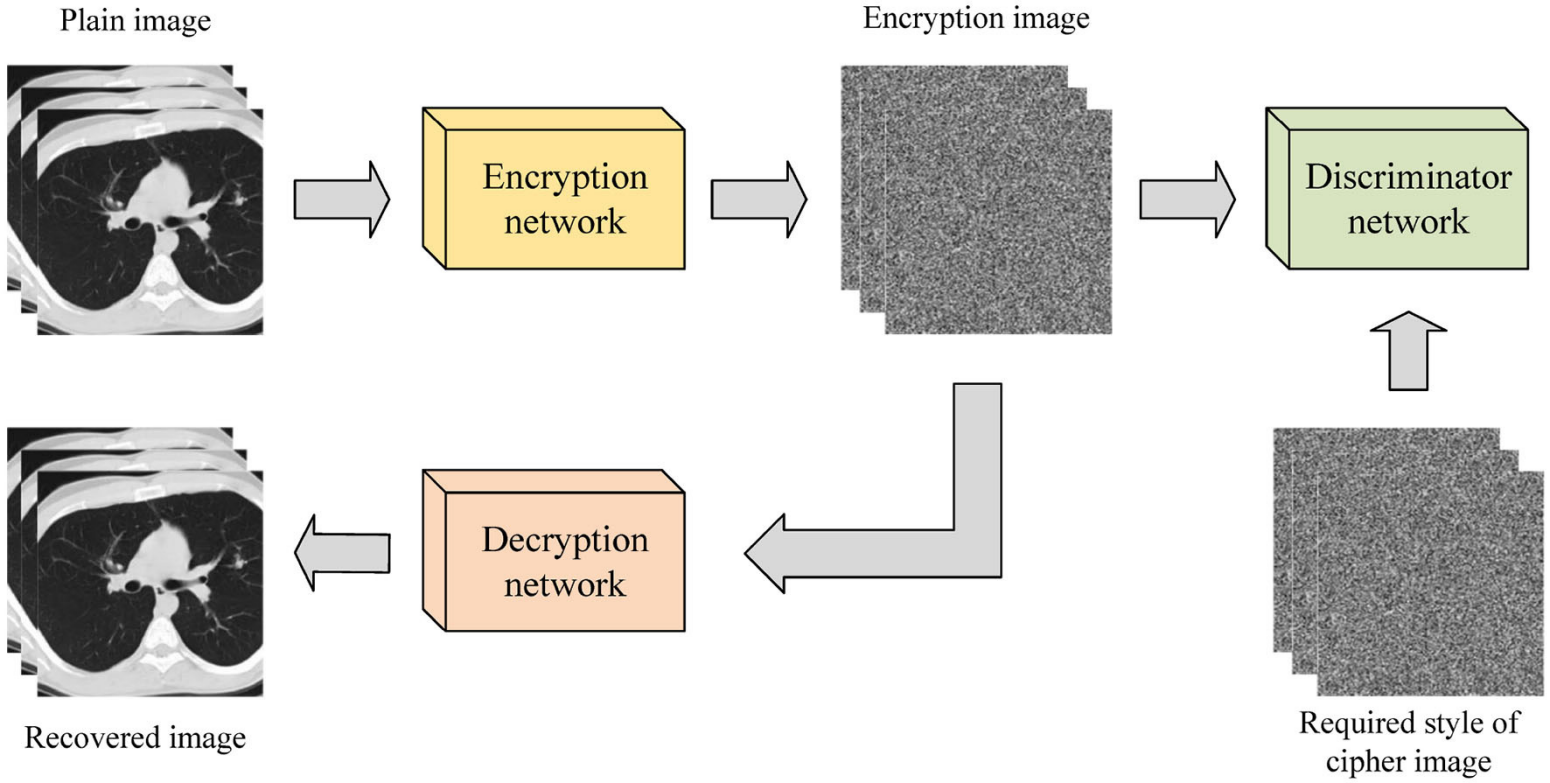
Image encryption and decryption process based on style transfer.

According to Panwar et al. (2023), existing image encryption systems based on deep learning can be categorized into three types: those based on style transfer, those based on enhanced diffusion characteristics, and those based on deep learning and chaotic systems. Image encryption systems based on style transfer aim to extract the style of ordinary images through the encryption network and convert them into the style of encrypted images through model training. This method often uses Generative adversarial network (GAN) and its variants to achieve the conversion of images from the original domain to the target domain. Figure 2 illustrates the image encryption and decryption process based on style transfer. Ding et al. (2020) proposed DeepEDN, an image encryption and decryption network based on deep learning, to encrypt and decrypt medical images. The network uses Cycle-generating adversarial networks (Cycle-GAN) as the main learning network and introduces a region of interest (ROI) mining network to extract objects of interest from encrypted images, achieving a better encryption effect. It was demonstrated that DeepEDN is more efficient and secure when tested on the chest X-ray dataset.

**Figure 3:**
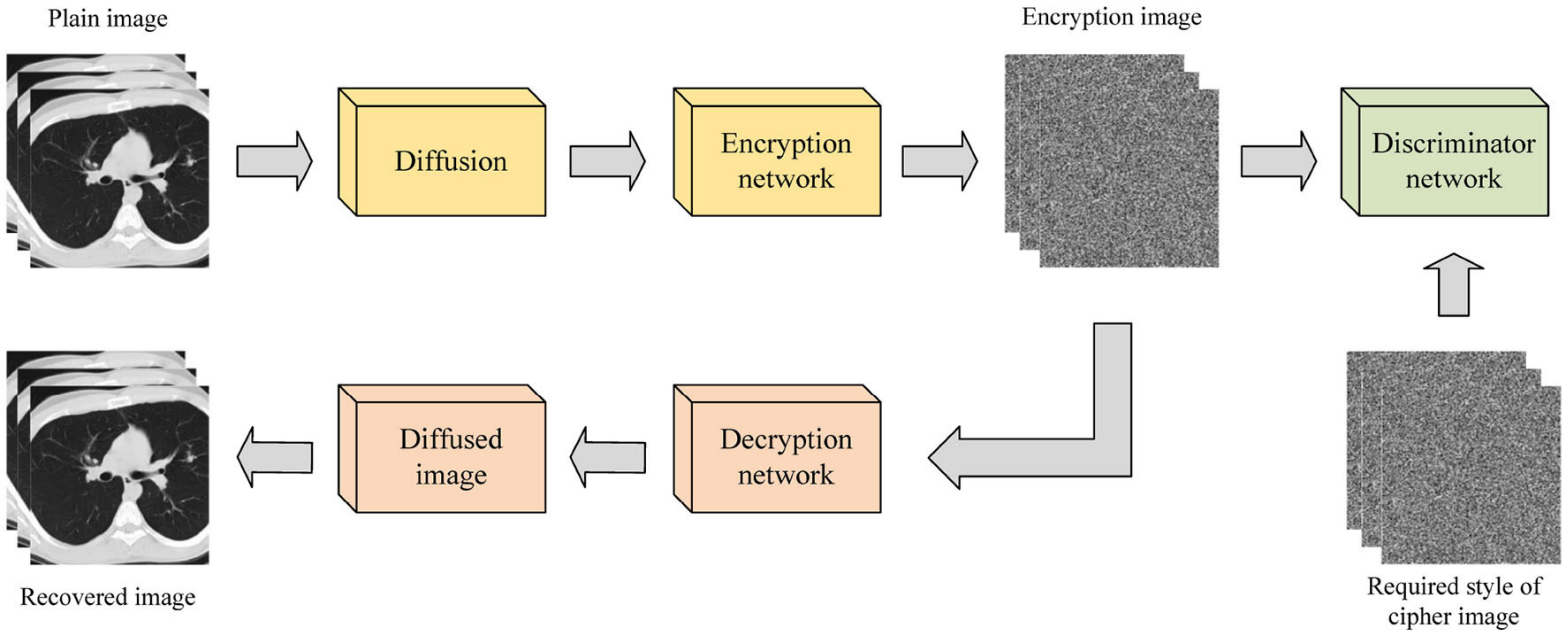
Image encryption process based on enhanced diffusion characteristics.

The image encryption system based on enhanced diffusion characteristics diffuses the original image before encrypting it with an encryption network. This method can also be implemented using GAN and its variants, as shown in Figure 3. Bao and Xue (2021) proposed a hybrid image encryption algorithm that combines a traditional diffusion algorithm with Cycle-GAN. The neural network is used to replace and slightly diffuse the image, followed by traditional diffusion algorithms to further diffuse the pixels. This addresses the weak avalanche effect of Cycle-GAN during the image encryption process. Experimental results demonstrate that the number of pixel change rate (NPCR) and the unified average change intensity (UACI) values reached 99.64 and 33.49%, respectively. Wang and Zhang (2022) proposed an image encryption algorithm based on a DNN. This method directly designs a new encryption unit with a deep neural network (EDNN) for encrypting the original image without training the network, followed by a decryption unit (DDNN) that is symmetrical to the EDNN structure for image decryption. Experimental results validate the effectiveness and security of EDNN in image encryption.

**Figure 4:**
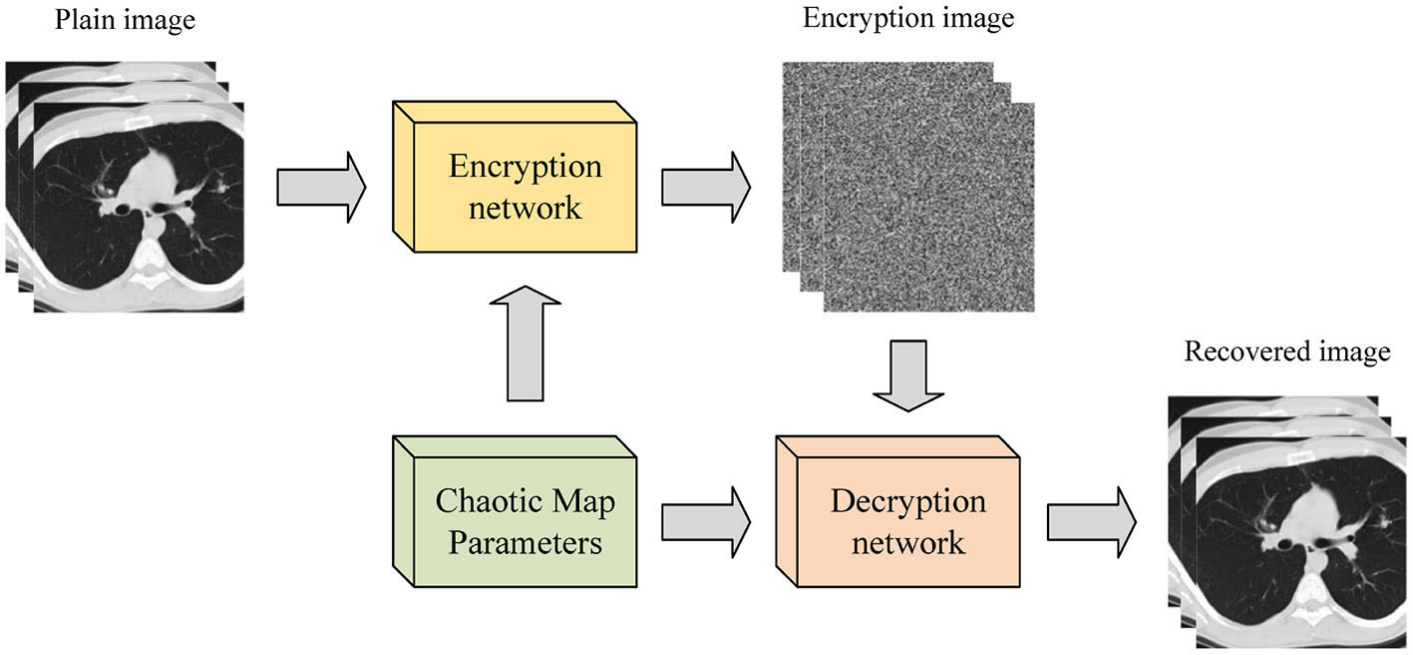
Image encryption process based on deep learning and chaotic system.

The combination of deep learning and chaotic systems enables image encryption through convolution of ordinary images, with the convolution kernel parameters updated by the chaotic sequence of a given chaotic map. This encryption process is depicted in Figure 4. Wu et al. (2021) introduced an image encryption method based on adversarial neural cryptography (ANC) and SHA control chaos. This approach obtains an intermediate image similar to noise by training a GAN, and subsequently performs an XOR operation based on the Logistic mapping on the intermediate image to generate the final ciphertext. Experimental results confirm the method's reliability and security. Chai et al. (2022) presented a robust compressed sensing image encryption algorithm using GAN, CNN denoising network, and chaotic systems. This approach acquires the measurement results of the original image through a sampling network, replaces them with the Logistic-Tent chaotic system to obtain the encrypted image, obtains the decryption measurement results by anti-replacement of the encrypted image, and obtains the decryption reconstruction image through the reconstruction network. Finally, CNN denoising improves the image quality (Fan et al., 2023). Experimental results demonstrate that this method has high reconstruction quality, robustness, and security. Compared with the other two image encryption methods, this method performs better in resisting plaintext attacks and has higher security (Cabán et al., 2022).

Although the combination of deep learning and image encryption is still in its infancy, the aforementioned studies have yielded promising results. However, with the increasing depth of neural networks, the issue of gradient disappearance can arise, posing a significant challenge to image encryption efficacy (Bao and Xue, 2021; Zhu et al., 2022). To address this issue, we propose a novel chaotic medical image encryption method that employs an attention mechanism fusion ResNet model. This method leverages the residual module to overcome the gradient disappearance problem and introduces an attention mechanism to enhance the model's focus on the region of interest, thereby improving encryption performance.

## 3. Methods

This section mainly introduces the algorithms used in this study, such as ResNet, attention mechanism, then clarifies the overall process of our proposed method, and finally gives some evaluation metrics for evaluating the quality of medical image encryption.

### 3.1. ResNet

ResNet is a special CNN model (He et al., 2016), which has strong feature extraction ability as traditional CNN, and solves the problem of gradient disappearance encountered by deep CNN by introducing residual module, which has a broader application prospect. Convolution layer, pooling layer and fully connected layer are also important components of ResNet (Wu et al., 2022).

The convolution layer performs feature extraction, and the deep analysis of the data is realized through the convolution kernel (Zhang et al., 2023). The convolution operation is very simple. The high-dimensional information can be obtained by multiplying and summing the convolution kernel with the feature information of the same size and adding the bias term (Li et al., 2023). Then the non-linear expression ability of the network is enhanced by the activation function. The convolution formula is expressed as:


(1) $$\begin{array}{l} c_{i}=f\left(w_{i}*x_{i}+b_{i}\right) \end{array}$$

where *x*_*i*_ denotes the input data, *w*_*i*_ denotes the weight matrix, *b*_*i*_ denotes the bias vector, * denotes the convolution operation, and *f* denotes the activation function.

The pooling layer can reduce the amount of data and parameters and reduce network complexity. Its operation process is roughly the same as convolution, the difference is that only the average or maximum value of the operation object is taken during the pooling operation. The pooling formula is shown in Equation (2).


(2) $$\begin{array}{l} p_{i}=h\left(c_{di+1-d},c_{di+2-d},...,c_{di+a-d}\right)+b_{i} \end{array}$$

where *d* denotes the step size, *a* denotes the window size, *b*_*i*_ denotes the bias vector, and *h* denotes the pooling function.

The fully connected layer is used to integrate the extracted feature information (Papadaki et al., 2022). The extracted feature information is flattened and input into the fully connected layer to obtain the corresponding output. The full connection formula is as follows.


(3) $$\begin{array}{l} y_{i}=f\left(w_{i}p_{i}+b_{i}\right) \end{array}$$

where *w*_*i*_ denotes the weight matrix, *b*_*i*_ denotes the bias vector, and *f* denotes the activation function.

**Figure 5:**
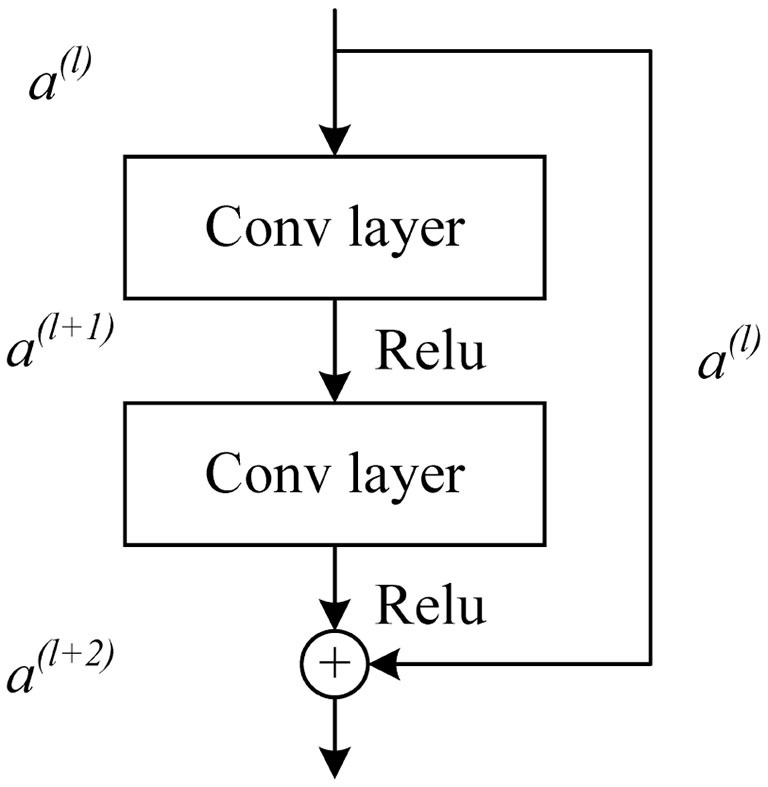
Basic residual unit of ResNet.

Unlike traditional CNN, ResNet is composed of multiple residual blocks in series, which can easily adjust the width and depth of the network to obtain networks with different expression capabilities and does not need to worry about the problem of gradient disappearance (Ning et al., 2020b). It has more powerful adaptive feature extraction capabilities for two-dimensional medical image data (Raman et al., 2021; Ahamed et al., 2023). The residual structure is shown in Figure 5.

The activation vector *a*^(*l*+1)^ of the next layer unit can be obtained by convolution and activation function operation of the activation vector *a*^(*l*)^ of the *l* layer unit. The activation vector *a*^(*l*+1)^ of the *l*+1 layer is added to the jump connected *a*^(*l*)^ after convolution operation, and then the output *a*^(*l*+2)^ of the residual block can be obtained by activation function operation. The formula is expressed as follows:


(4) $$\begin{array}{l} a^{\left(l+1\right)}=f\left(w^{\left(l+1\right)}*a^{\left(l\right)}+b^{\left(l+1\right)}\right) \end{array}$$

(5) $$\begin{array}{l} a^{\left(l+2\right)}=f\left(w^{\left(l+2\right)}*a^{\left(l+1\right)}+b^{\left(l+2\right)}+a^{\left(l\right)}\right) \end{array}$$

### 3.2. Attention mechanism

**Figure 6:**
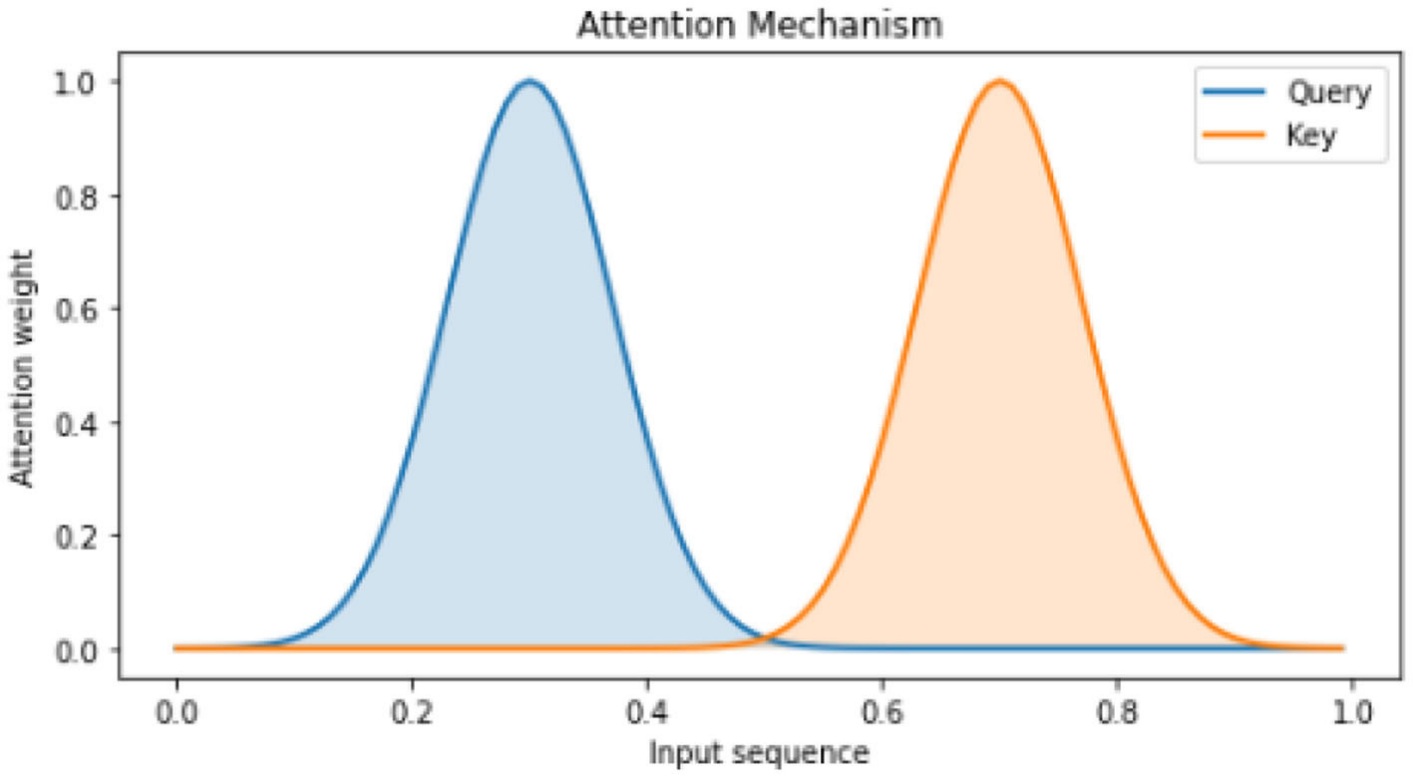
Visualization of attention mechanism.

In the field of natural language processing, attention mechanisms have gained significant popularity in recent years. These mechanisms have emerged as effective tools for various tasks, allowing models to selectively concentrate on specific segments of the input sequence (Ning et al., 2020c). By assigning weights to individual elements, attention enables the model to discern the relative significance of each component during the prediction process (Ning et al., 2020a). This enhanced focus on relevant information has proven valuable in improving the performance and accuracy of natural language processing systems. Figure 6 illustrates a simple attention mechanism, where two Gaussian distribution curves are used to simulate the Query and Key. The shading technique used in the figure is the fill between function, which effectively illustrates the attention weights of each curve (He and Ye, 2022). By assigning darker colors to areas with higher attention weights and lighter colors to areas with lower attention weights, the figure visually represents the essence of the attention mechanism (Chen et al., 2023). This mechanism aims to improve sequence learning by assigning varying weights to different parts of the input sequence (Saiki et al., 2023).

**Figure 7:**
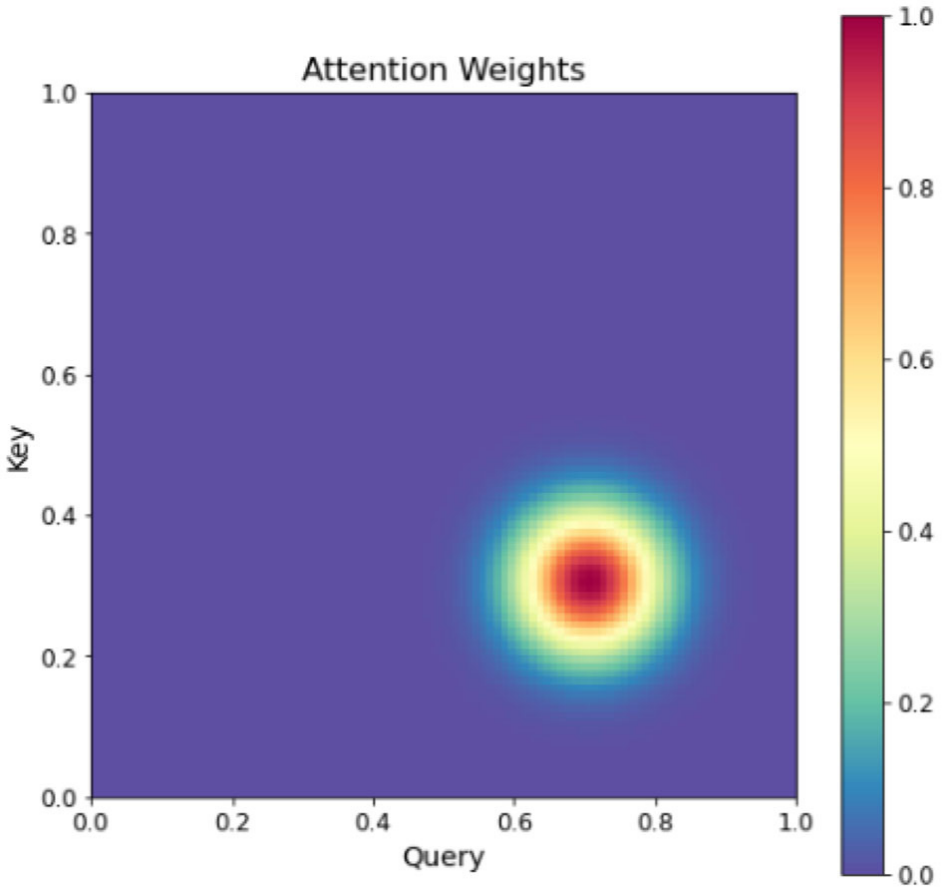
Heatmap of attention weights.

In Figure 7, a more intricate attention mechanism is depicted through a two-dimensional matrix that showcases the attention weights between Query and Key pairs. Each element in the matrix corresponds to the attention weight between a specific Query and Key pair (Van Hooren et al., 2023). By utilizing darker colors for higher attention weights and lighter colors for lower attention weights, the figure provides a clear visualization of the varying degrees of attention (Yi et al., 2023). This visualization effectively demonstrates the dynamic allocation of attention weights within the input sequence, facilitating enhanced sequence learning.

This figure provides a more intuitive understanding of the attention mechanism, which assigns different weights to different parts of the input sequence to achieve better sequence learning. The double attention mechanism is an extension of the standard attention mechanism, which incorporates both query-level and key-level attentions to better capture the relationships between the input and output. Specifically, given a set of input vectors *x*_1_, *x*_2_, ..., *x*_*n*_ and a set of output vectors *y*_1_, *y*_2_, ..., *y*_*m*_, the double attention mechanism first computes the query-level attention weights *a*_*q*_ and key-level attention weights *a*_*k*_ as follows:


(6) $$\begin{array}{l} a_{q}=\text{softmax}\left(W_{q}q\right) \end{array}$$

(7) $$\begin{array}{l} a_{k}=\text{softmax}\left(W_{k}k\right) \end{array}$$

where *q* and *k* are learnable query and key vectors, and *W*_*q*_ and *W*_*k*_ are learnable weights. The query-level attention weights are used to compute the context vector *c*_*q*_ as a weighted sum of the input vectors:


(8) $$\begin{array}{l} c_{q}=\overset{n}{\underset{i=1}{\sum }}a_{q}^{i}x_{i} \end{array}$$

Similarly, the key-level attention weights are used to compute the context vector *c*_*k*_ as a weighted sum of the output vectors:


(9) $$\begin{array}{l} c_{k}=\overset{m}{\underset{j=1}{\sum }}a_{k}^{j}y_{j} \end{array}$$

The final output vector *y* is obtained by concatenating the context vectors *c*_*q*_ and *c*_*k*_ and passing them through a linear layer:


(10) $$\begin{array}{l} y=\text{ReLU}\left(W_{o}\left[c_{q};c_{k}\right]\right) \end{array}$$

The channel attention mechanism is an important component of convolutional neural networks (CNNs) that enables the encoding of distinct object features on separate channels within the convolutional feature map. This mechanism, dynamically adjusts the weights assigned to each channel during the learning process.

By assigning weights to individual channels, a vector is generated, with each element representing the weight associated with a specific channel in the feature map. This vector guides the network's attention toward specific regions of interest within the pedestrian being analyzed. To illustrate the implementation of the attention mechanism, Algorithm 1 presents a pseudo-code representation.

**Algorithm 1:** The attention mechanism algorithm pseudo-code.

**Input:**AttentionMechanismG(V;E), node features
$\left\{x_{v},\forall v\in V\right\}$, Number of layers *K*,
Attention mechanism *a*, Trainable parameters Θ for neural networks
**Output:** Node embeddings **h**_*v*_ for all v∈V
1: for each node v∈V do
2: N(v)← the set of neighbors of *v* in G
3: ${\text{h}}_{v}^{\left(0\right)}\leftarrow {\text{x}}_{v}$
4: for *k* = 1 to *K* do
5: ${\text{h}}_{v}^{\left(k\right)}\leftarrow {\text{AGGREGATE}}^{\left(k\right)}\left({\text{h}}_{u}^{\left(k-1\right)}:u\in N\left(v\right)\right)$
6: ${\text{h}}_{v}^{\left(k\right)}\leftarrow {\text{COMBINE}}^{\left(k\right)}\left({\text{h}}_{v}^{\left(k-1\right)},{\text{h}}_{v}^{\left(k\right)}\right)$
7: ${\text{h}}_{v}^{\left(k\right)}\leftarrow {\text{ATTEND}}^{\left(k\right)}\left({\text{h}}_{u}^{\left(k\right)}:u\in N\left(v\right),{\text{h}}_{v}^{\left(k\right)};\Theta ,a\right)$
8: ${\text{h}}_{v}\leftarrow {\text{h}}_{v}^{\left(K\right)}$ for each node v∈V

The utilization of the channel attention mechanism enhances the network's ability to focus on relevant and discriminative features, thus improving its performance in various computer vision tasks. It provides a mechanism for adaptively recalibrating the importance of different channels, enabling more effective information processing within CNNs (Gao et al., 2023).

Overall, the channel attention mechanism plays a crucial role in optimizing the representation and learning capabilities of CNNs, enabling them to extract and emphasize relevant features for improved object recognition and classification (Dewi et al., 2023).

where *Q*, *K*, and *V* represent query vectors, key vectors, and value vectors, respectively, *d*_*k*_ represents the dimension of the key vector, and *n* represents the number of key vectors. The algorithm first obtains the attention output *O* by calculating the attention score α_*i*_ to weighted the sum of the value vectors. The attention score α_*i*_ is calculated based on the dot product between the query vector and the key vector, scaled by $\sqrt{d_{k}}$, and then normalized by softmax.

**Figure 8:**
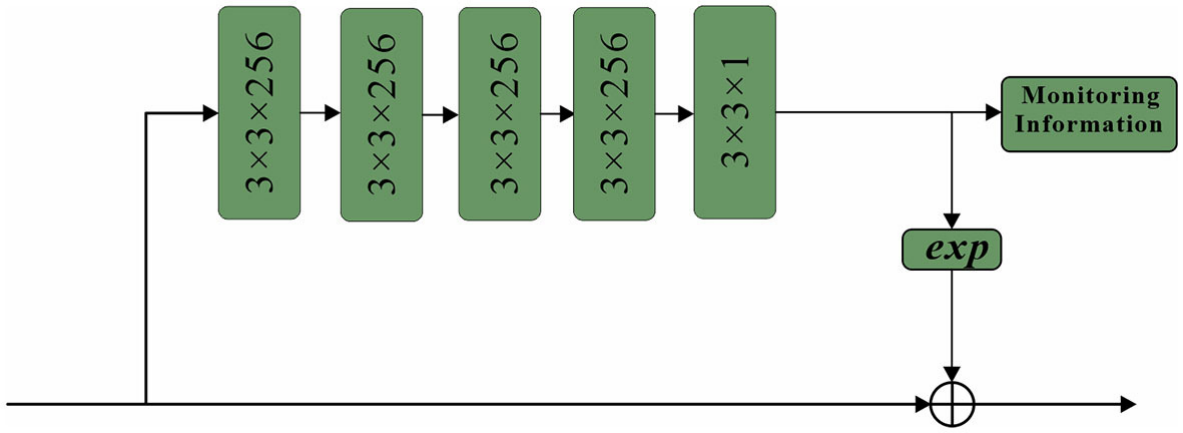
Channel attention sub-network structure.

The network architecture, as depicted in Figure 8, employs classification branches that undergo initial pooling. The resulting pooled weight vectors are then passed through fully connected layers FC1 and FC2, which perform “compression” and “stretching” operations, respectively. To confine the vector components within the range of 0 and 1, a sigmoid function is applied. Subsequently, the two vectors are combined and fused to produce the final weight vector. In order to capture both prominent features and average characteristics across channels, a combination of global pooling and maximum pooling is utilized (Zhang Y.-H. et al., 2022). This approach allows the network to prioritize visible regions of pedestrians while retaining overall channel information (Zhang M. et al., 2022). Moreover, the channel attention module plays a crucial role in generating a channel attention map by leveraging inter-channel relationships among features. By assigning higher weights to channels exhibiting strong responses to salient targets, this module effectively highlights relevant information. Figure 8 presents a schematic representation of the channel attention module's structure. This design enables the network to enhance its discriminative power by attending to significant features and channel-level details, resulting in improved pedestrian detection performance.

To begin, the input feature F undergoes both maximum pooling and average pooling operations to extract essential information while reducing redundancy. The resulting aggregated feature map, which encapsulates interval information, is subsequently fed into a shared network. This network compresses the spatial dimension of the input feature map by summing the elements within each feature map, generating channel attention weights. The calculation formula for obtaining these weights is expressed as follows:


(11) $$\begin{array}{l} L_{r}\left(t,t^{*}\right)=\underset{n\in A}{\sum }\left(p_{n}^{*}=1\right)\underset{i\in x,y,w,h}{\sum }smoothL1\left(t_{i}^{n}-t_{i}^{*n}\right) \end{array}$$

**Figure 9:**
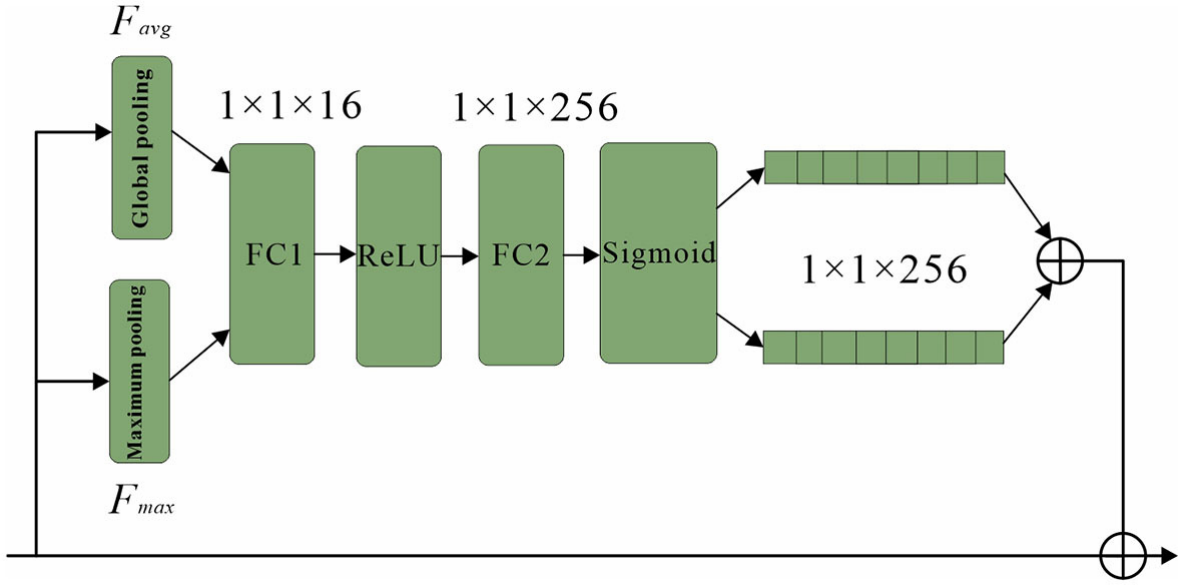
Structure of spatial attention sub-network.

The paper introduces a network architecture called spatial attention, which utilizes a mask of the same size as the original image features. This mask assigns weights to each element of the feature map, indicating the importance of that pixel's corresponding region. The network continuously learns and adjusts these weights, enabling it to focus on specific regions. The sub-network structure of the spatial attention mechanism employed in this study is depicted in Figure 9. Thus, the essential concept remains unaltered while minimizing repetition.

The proposed approach begins with the application of a series of convolutional layers to the initial feature map. Specifically, four convolutional layers, each with a size of 3 × 3 and 256 channels, are utilized to process the feature map (Vidhya et al., 2020). Subsequently, a single mask is generated by employing a 3 × 3 convolutional layer with 1 channel, effectively compressing the intermediate results. This mask plays a crucial role in preserving the underlying background information and adjusting the weights associated with each position within the feature map.

To enhance the learning process of the spatial attention mechanism, a supervised approach is adopted, where the spatial attention mechanism is trained using pedestrian information as labels (Noda et al., 2023). The labels are assigned at the pixel level, with specific values assigned to different regions of interest. In particular, pixel values within the visible region of a pedestrian's bounding box are set to 1, while those within the full-body bounding box are set to 0.8. Background regions are assigned a value of 0. This labeling scheme guides the spatial attention mechanism to focus its attention primarily on the road regions within the input frame, particularly emphasizing the visible sections. In conclusion, the proposed double attention mechanism combines query-level and key-level attentions to capture the intricate relationships between input and output sequences more effectively. By selectively attending to specific parts of the sequences, the model can discern the importance of each element when making predictions, leading to improved performance and better overall results.

### 3.3. Overall framework of AT-ResNet-CM

**Figure 10:**
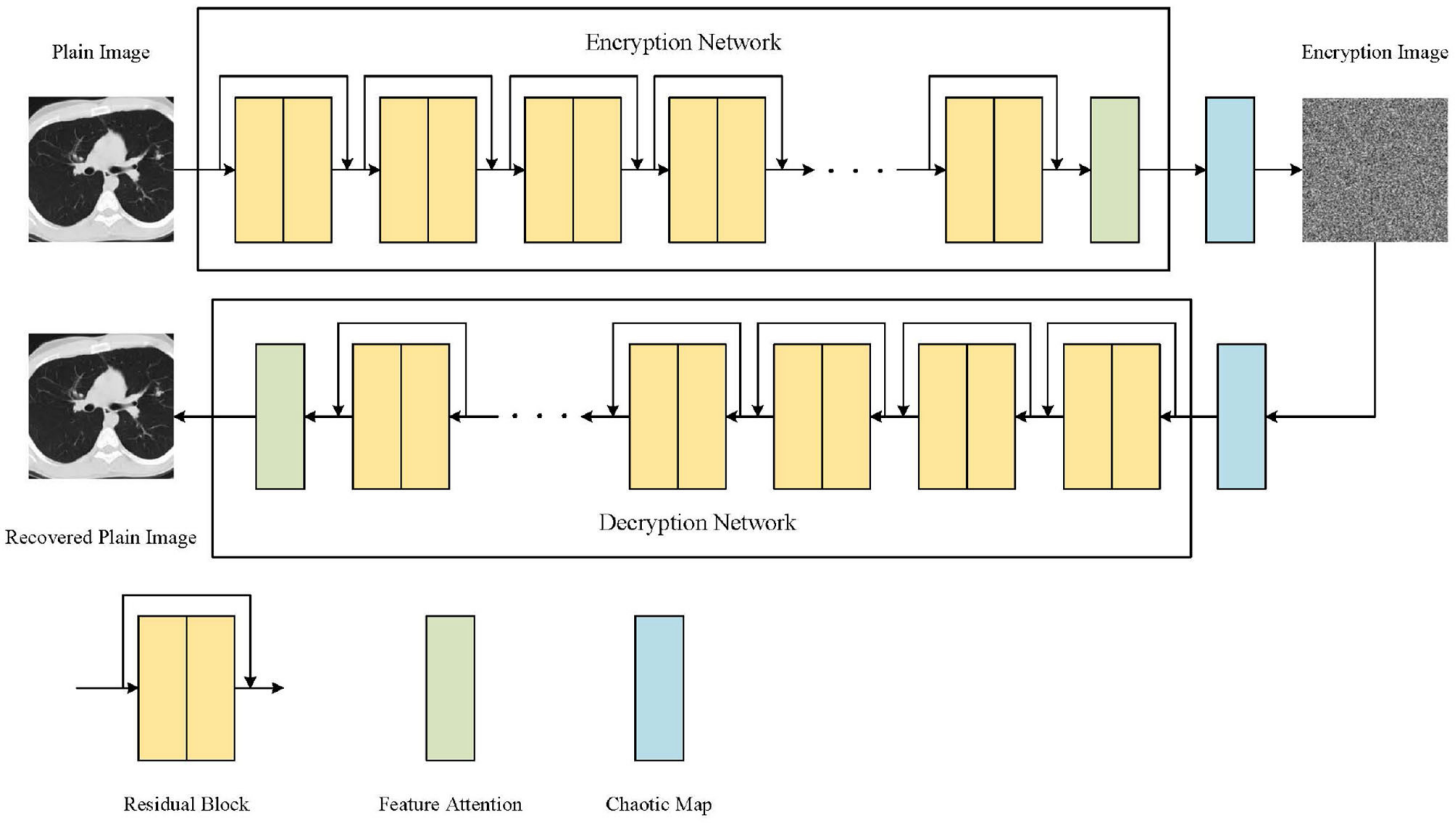
The workflow of our proposed medical image encryption method.

The proposed medical image encryption method, as depicted in Figure 10, encompasses a comprehensive workflow. The method primarily consists of three key components: ResNet, chaotic mapping, and the attention module. ResNet, known for its deep structure, plays a crucial role in capturing the intricate and high-dimensional feature expressions present in medical images. By leveraging the power of ResNet, the method can extract richer and more informative features, enabling a more comprehensive understanding of the image content. Moreover, ResNet's unique skip or jump connections facilitate faster convergence of the model during the training process. These connections allow the model to learn from both shallow and deep layers simultaneously, enhancing its capability to extract relevant features and improving overall performance. To further enhance the encryption quality of the medical image, the encryption results obtained from ResNet undergo additional processing using a Logistic chaotic system. This chaotic mapping process adds an extra layer of security and randomness to the encryption process, making it more challenging for unauthorized individuals to decipher the encrypted image. By leveraging the inherent chaotic nature of the Logistic map, the method achieves enhanced encryption strength and resilience against attacks. Additionally, the attention module plays a crucial role in guiding ResNet's focus toward the regions of interest within the medical image. By employing attention mechanisms, the model can effectively allocate its computational resources to important areas, such as regions containing critical medical information or abnormalities. This targeted focus improves the encryption performance of the model, ensuring that significant details are appropriately encrypted and safeguarded. As a result, the method not only enhances security but also improves the reliability and effectiveness of the encryption process. In summary, the proposed medical image encryption method integrates ResNet, chaotic mapping, and an attention module to achieve robust encryption. By leveraging ResNet's deep structure, the method captures essential features, while the chaotic mapping process enhances encryption quality. The attention module ensures that important regions receive appropriate focus, thereby improving overall encryption performance and making the method more secure and reliable. The workflow of the method outlined in Figure 10 demonstrates how these components synergistically contribute to the encryption process.

The medical image encryption method in this paper is mainly realized by some convolution layers on ResNet. The deep feature extraction of the original medical image is carried out by multi-layer residual blocks, and the similarity between the extracted features and the target is calculated by the attention module, so that the model can pay more attention to the relevant information and ignore the irrelevant information, so as to improve the model's ability to extract important features. Finally, the encrypted image is obtained by XOR operation based on Logistic chaotic system, which is the whole process of medical image encryption. The decryption process of the encrypted medical image is to obtain the intermediate result by performing the XOR operation based on the Logistic chaotic system on the encrypted image, and then the decrypted image can be obtained by the ResNet model with the attention mechanism.

### 3.4. Evaluation methods

The experiment uses histogram analysis, correlation analysis, and information entropy analysis to evaluate the medical image encryption effect of this method.

Histogram analysis is an important index to test the medical image encryption method, which can visually display the statistical characteristics of each gray pixel of the encrypted image. The attacker can easily obtain the statistical information of the original image from the non-uniform histogram. Usually, the more uniform the gray histogram of the encrypted image is, the more difficult it is for the attacker to obtain the relevant information of the original image from the encrypted image.

Correlation analysis is a deep mining of the intrinsic correlation between two or more variables, so as to measure the correlation between variables. For general digital images, it has a strong correlation between adjacent pixels in the four directions of horizontal, vertical, diagonal, and anti-diagonal. Whether this correlation can be effectively broken is an important indicator for evaluating a medical image encryption method. Usually, the smaller the correlation between adjacent pixels, the better the medical image encryption effect. The calculation process of the correlation coefficient is expressed by the formula:


(12) $$\begin{array}{l} cov\left(x,y\right)=\frac{1}{N}\overset{N}{\underset{i=1}{\sum }}\left(x_{i}-E\left(x\right)\right)\left(y_{i}-E\left(y\right)\right) \end{array}$$

(13) $$\begin{array}{l} PCC=\frac{cov\left(x,y\right)}{\sqrt{D\left(x\right)}\sqrt{D\left(y\right)}} \end{array}$$

where *x*_*i*_ and *y*_*i*_ are adjacent pixels, *E*(*x*) and *E*(*y*) are the expectations of corresponding pixels, and *D*(*x*) and *D*(*y*) are the variances of corresponding pixels.

In the field of digital image encryption, information entropy can reflect the uncertainty of an image. Generally, the larger the information entropy of the encrypted image, the greater the uncertainty of the information it contains, that is, the higher the security of the encrypted image. For the 8-bit gray level encrypted image, the ideal value of information entropy is 8. The calculation formula of information entropy is as follows:


(14) $$\begin{array}{l} H=-\overset{L}{\underset{i=0}{\sum }}p\left(i\right)\log_{2}p\left(i\right) \end{array}$$

where *L* represents the gray level of the medical image, and *p*(*i*) is the probability of gray value *i*.

## 4. Experimentation

This section introduces the relevant information of the data set and experimental parameter settings used in the experiment, and then gives the chaotic medical image encryption experimental results of the attention mechanism fusion ResNet model. The security and flexibility of the AT-ResNet-CM model in medical image encryption are evaluated by relevant evaluation methods and compared with other models to verify the superiority of the AT-ResNet-CM model.

### 4.1. Dataset description and experimental parameter settings

In clinical applications, medical images mainly include X-ray images, CT, magnetic resonance imaging and other types. The experimental data used in this paper are from the AMRG Cardiac MRI Atlas dataset and the COVID-19 Chest X-ray dataset. All medical images are grayscale images, and the images size are adjusted to 512 × 512. These medical images are used to evaluate the performance of the AT-ResNet-CM model.

The experiments in this paper are carried out on Windows11 64-bit operating system, Intel Core i7-10870 CPU @ 2.20 GHz, 16 GB running memory, Nvidia GeForce RTX 2070 with Max-Q Design graphics card using TensorFlow deep learning platform and Python programming language. The experimental parameters are set as shown in Table 1.

**Table 1:** Experimental parameter settings.

**Parameter**	**Setting**
Learning rate	0.0001
Batch size	64
Epoch	300
Optimizer algorithm	Adam
Loss function	MSE

### 4.2. Medical image encryption experiment of ResNet-CM model

**Figure 11:**
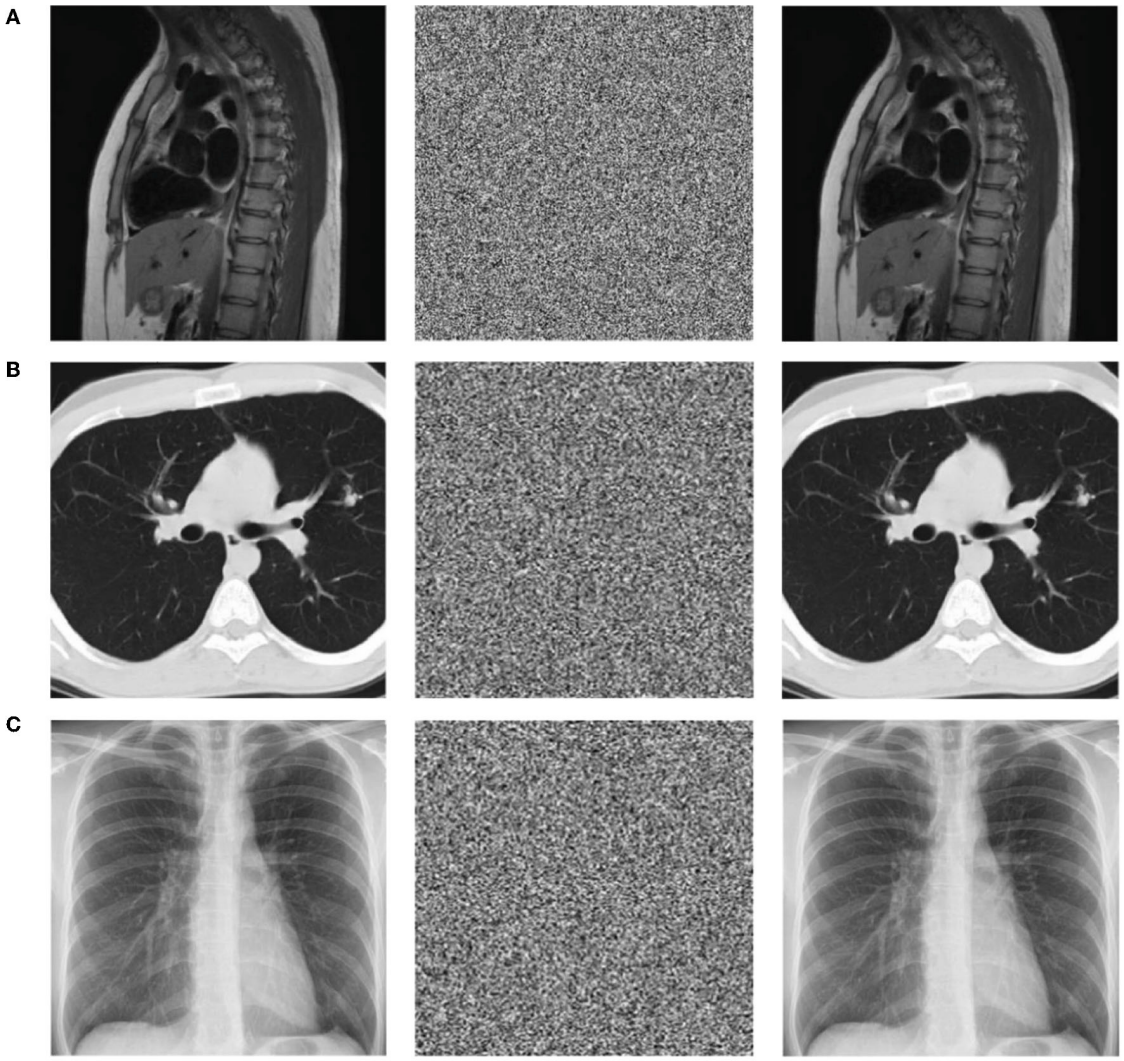
The results display of the original image (left), encrypted image (middle), and decrypted image (right). **(A)** Chest medical image. **(B)** Brain medical image. **(C)** Lung medical image.

**Figure 12:**
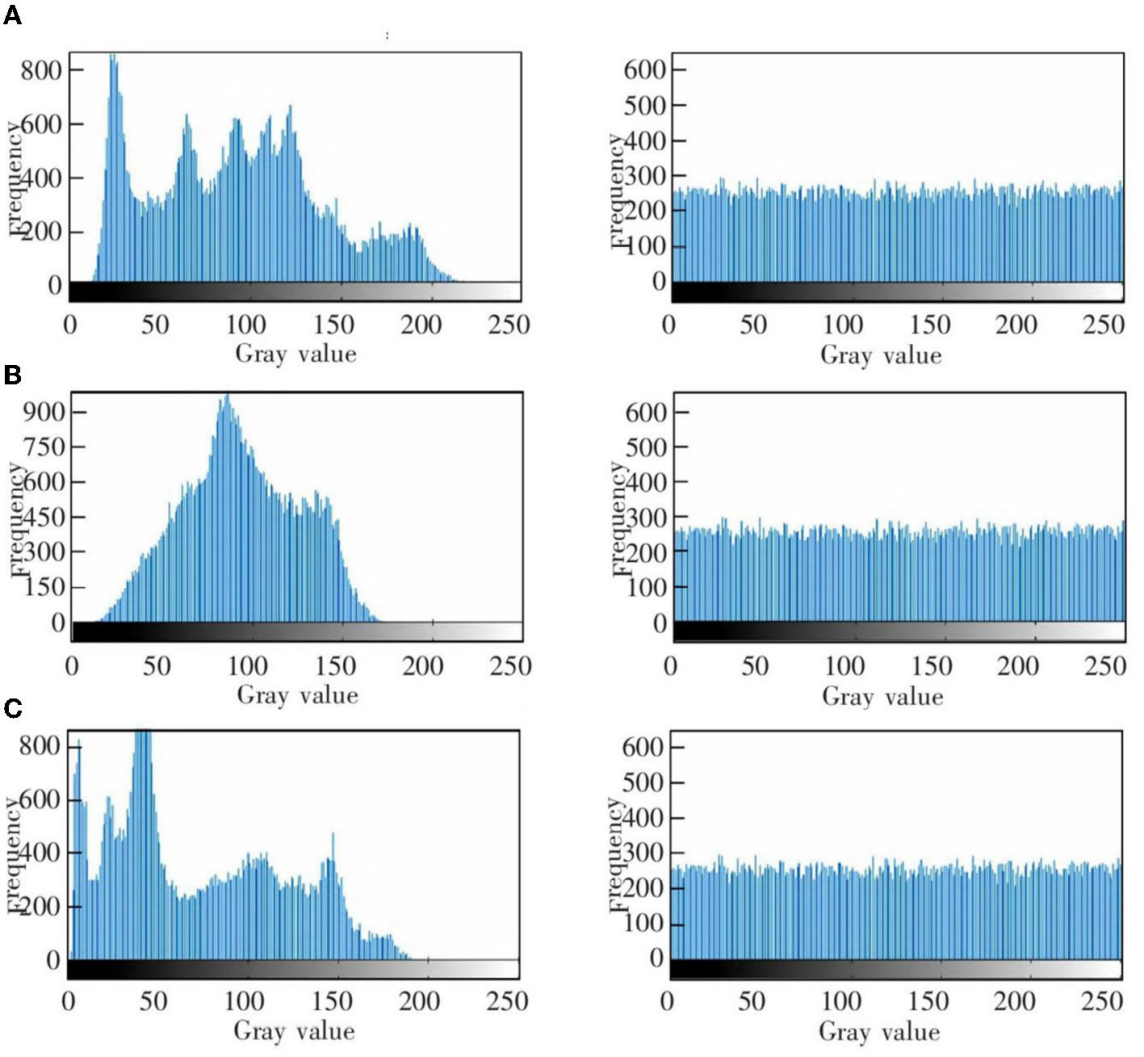
The gray histogram of the original image (left) and encrypted image (right). **(A)** Chest medical image. **(B)** Brain medical image. **(C)** Lung medical image.

**Figure 13:**
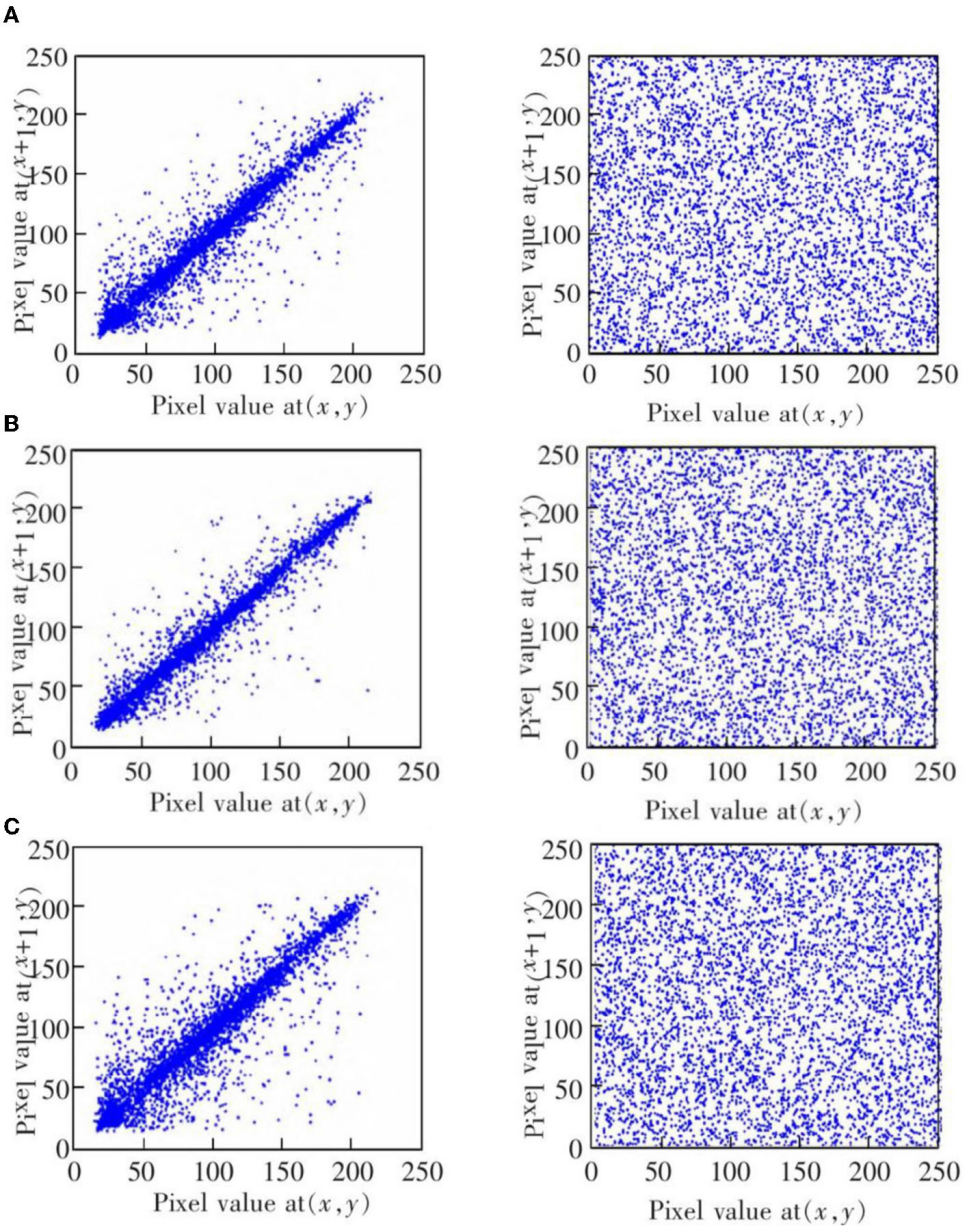
The horizontal correlation map of the original image (left) and encrypted image (right). **(A)** Chest medical image. **(B)** Brain medical image. **(C)** Lung medical image.

This experiment is mainly used to test the encryption and decryption effects of ResNet-CM model on Chest, Brain, and Lung medical images. The experimental results are shown in Figure 11. To better observe and measure the medical image encryption effect of the ResNet-CM model, the gray histogram of the original image and the encrypted image is shown in Figure 12, and the horizontal pixel correlation diagram of the original image and the encrypted image is shown in Figure 13. The horizontal correlation coefficient and information entropy of the original image and the encrypted image are shown in Tables 2, 3, respectively.

**Table 2:** The horizontal correlation coefficient of the original and encrypted medical images.

**Image name**	**Original image**	**Encrypted image**
Chest medical image	0.9625	0.0021
Brain medical image	0.9372	−0.0064
Lung medical image	0.9756	0.0019

**Table 3:** The information entropy of the original and encrypted medical images.

**Image name**	**Original image**	**Encrypted image**
Chest medical image	7.3863	7.9887
Brain medical image	6.9735	7.9658
Lung medical image	7.2492	7.9911

Figures 11A–C shows the encryption and decryption results of Chest, Brain, and Lung medical images, respectively. The first column is the original image, the second column is the encrypted image, and the third column is the decrypted image. Figures 12A–C are the gray histograms of the original and encrypted medical images of Chest, Brain, and Lung, respectively. The abscissa is the gray value of the medical image, and the ordinate is the frequency of the gray value. The gray histogram distribution of these original medical images has peaks and troughs, and even a completely blank gray interval appears. The distribution of the encrypted image on the gray histogram is very uniform and smooth, and the probability of the pixel intensity value of each gray interval is basically similar. Figures 13A–C is the horizontal pixel correlation map of the original and encrypted medical images of Chest, Brain, and Lung. The abscissa is the pixel value at (*x, y*) and the ordinate is the pixel value at (*x*+1, *y*). It can be seen that the adjacent pixels of these original medical images are roughly distributed on the diagonal of the correlation map, while the adjacent pixels of the encrypted image are relatively evenly distributed in the whole plane of the correlation map.

Table 2 compares the horizontal correlation coefficients of the original and encrypted medical images of Chest, Brain, and Lung. The horizontal correlation coefficients of the original medical images of Chest, Brain, and Lung reached 0.9625, 0.9372 and 0.9756, respectively, which are very close to 1. In contrast, the horizontal correlation coefficients of Chest, Brain, and Lung medical images encrypted by this method have been greatly reduced, which are 0.0021, −0.0064, and 0.0017, respectively, which are very close to 0. Table 3 compares the information entropy of the original and encrypted medical images of Chest, Brain and Lung. The information entropy of the original medical images of Chest, Brain and Lung reached 7.3863, 6.9735, and 7.2492, respectively, and there is a certain distance from the ideal value 8. In contrast, the information entropy of Chest, Brain, and Lung medical images encrypted by this method has been greatly improved, which are 7.9887, 7.9658, and 7.9911, respectively, which are very close to the ideal value 8.

### 4.3. Comparative experiment of different models

To highlight the ResNet-CM model has better medical image encryption effect than other models, we carried out comparative experiments between ResNet-CM model and Zhang et al. (2012), Ding et al. (2020), Yang et al. (2020), Hua et al. (2021), Wu et al. (2021), Wang et al. (2022), and Zhu et al. (2022). To better measure the medical image encryption effect of these models, the horizontal correlation coefficient and information entropy of these models on encrypted images are shown in Table 4.

**Table 4:** Comparison of horizontal correlation coefficient and information entropy of different models.

**Reference**	**Horizontal correlation coefficient**	**Information entropy**
Hua et al. (2021)	0.0283	7.1165
Zhang et al. (2012)	0.0259	7.1548
Yang et al. (2020)	0.0188	7.2831
Wang et al. (2022)	0.0135	7.6369
Zhu et al. (2022)	0.0089	7.7532
Ding et al. (2020)	0.0176	7.3582
Wu et al. (2021)	0.0065	7.8364
Ours	0.0021	7.9887

Table 4 is the horizontal correlation coefficient and information entropy comparison of ResNet-CM and other models on encrypted images. It can be seen that the horizontal correlation coefficients of ResNet-CM, Zhang et al. (2012), Ding et al. (2020), Yang et al. (2020), Hua et al. (2021), Wu et al. (2021), Wang et al. (2022), and Zhu et al. (2022) models on encrypted images are 0.0021, 0.0283, 0.0259, 0.0188, 0.0135, 0.0089, 0.0176 and 0.0065, respectively, which are relatively close to 0, and the information entropy reach 7.9887, 7.1165, 7.1548, 7.2831, 7.6369, 7.7532, 7.3582, and 7.8364, respectively, which are relatively close to the ideal value 8.

### 4.4. Comparative experiment of AT-ResNet-CM and ResNet-CM models

To analyze the influence of the introduction of attention mechanism on the effect of medical image encryption, we carried out medical image encryption experiments based on the AT-ResNet-CM model and compared it with the ResNet-CM model without attention mechanism. The horizontal correlation coefficient and information entropy of the two models on the encrypted image are shown in Table 5.

**Table 5:** The information entropy of the original and encrypted medical images.

**Model**	**Horizontal correlation coefficient**	**Information entropy**
Before	0.0021	7.9887
After	0.0010	7.9965

Table 5 shows the horizontal correlation coefficient and information entropy of AT-ResNet-CM and ResNet-CM models on encrypted images. The horizontal correlation coefficients of AT-ResNet-CM and ResNet-CM models are 0.0010 and 0.0021, respectively, which are very close to 0, and the information entropy reach 7.9965 and 7.9887, respectively, which are very close to the ideal value 8.

The capacity of the key space is also an important consideration to ensure the security of the encryption scheme. For an encryption scheme, the more cases the key can be selected, the higher the security of the scheme. Generally speaking, the basic requirement of key space is that when *S*>2^100^, the key system can resist brute-force attack (Hu et al., 2017). In the encryption algorithm of this paper, for the encryption part, the target random image is the sum of parameters represented by 8-bit binary. In addition to the key space of the chaotic system, the encryption key space and decryption key space of this method are at least 2^8 × 256 × 256^>2^100^, which can achieve a higher level of security. Therefore, the key space of this algorithm can effectively resist exhaustive attacks.

## 5. Discussion

This section mainly discusses the experimental results and analyzes the limitations of this work. In order to verify the effect of AT-ResNet-CM model in medical image encryption, we carried out medical image encryption experiment based on ResNet-CM model, comparative experiment between ResNet-CM model and other models, and comparative experiment between AT-ResNet-CM and ResNet-CM models, respectively. From the aspects of gray histogram analysis, adjacent pixel correlation analysis, information entropy analysis and key space analysis, it is fully proved that the encryption performance of this method is more superior and safer.

In the medical image encryption experiment based on ResNet-CM model, we can intuitively see from the medical image encryption and decryption results of Figure 11 that there is no similarity between the encrypted image and the original image, while the decrypted image is basically the same as the original image, indicating that the method performs well in medical image encryption and decryption. Figure 12 shows that the original medical image has strong characteristics, and the encrypted image breaks the statistical characteristics of the original image, which shows that the encrypted image can better resist the attack of statistical analysis. Figure 13 shows that there is a high correlation between the adjacent pixels of the original medical image, which means that when we take the pixel value of a point, the pixel value of its surrounding points is very close to its size. The adjacent pixels of the encrypted image are scattered in various regions, indicating that there is no connection between adjacent pixels, so it is impossible to infer the size range nearby according to the pixel value of the point. It can be seen from Table 2 that the horizontal correlation coefficient of the original medical images are close to 1, especially the Chest and Lung medical images are above 0.96, which means that the original medical image contains rich information. The horizontal correlation coefficient of the encrypted images are close to 0, indicating that the encryption algorithm in this paper effectively reduces the correlation between adjacent pixels. It can be seen from Table 3 that the information entropy of the original medical images are about 7, while the information entropy of the encrypted images are very close to the ideal value 8, showing higher uncertainty, indicating that the encryption algorithm in this paper makes the pixel values in each interval tend to be smooth, so that its distribution characteristics do not have weaknesses. In summary, the ResNet-CM model has sufficient security in medical image encryption.

In the comparative experiment of ResNet-CM model and other models, Table 4 shows that the horizontal correlation coefficients of these models are ranked from small to large as ResNet-CM, Zhang et al. (2012), Ding et al. (2020), Yang et al. (2020), Hua et al. (2021), Wu et al. (2021), Wang et al. (2022), amd Zhu et al. (2022), and the correlation coefficient of ResNet-CM model is very close to 0. Usually the smaller the correlation coefficient, the better the encryption effect. The information entropies of these models are ranked from large to small as ResNet-CM, Zhang et al. (2012), Ding et al. (2020), Yang et al. (2020), Hua et al. (2021), Wu et al. (2021), Wang et al. (2022), and Zhu et al. (2022), and the information entropy of the ResNet-CM model tends to an ideal value of 8. It can be seen that the ResNet-CM model performs better on two metrics, indicating that the ResNet-CM model breaks the statistical characteristics of the original medical image, and the encrypted image has less correlation between adjacent pixels and higher uncertainty, which can better resist the attack of statistical analysis. In summary, compared with the BP, CNN, ResNet, and other models used by other authors, the medical image encryption effect of ResNet-CM model is more significant.

The analysis shows that the unique characteristics of CNN model, such as parameter sharing and local connection, make it very superior in feature extraction, which is far from the BP model, so the encryption effect of BP model is the worst. The residual structure of the ResNet model solves the problem of gradient disappearance faced by deep CNN. The deeper network structure makes its non-linear expression ability more prominent, so the encryption performance of the ResNet model is better than that of the CNN model. The pseudo-randomness and unpredictability of chaotic system make it widely used in medical image encryption tasks. Combining deep learning with chaotic system for medical image encryption obviously has higher feasibility and security, so ResNet-CM model has achieved better encryption effect.

In the comparative experiment of AT-ResNet-CM and ResNet-CM models, Table 5 shows that compared with the ResNet-CM model, the horizontal correlation coefficient of the encrypted image of the AT-ResNet-CM model is reduced by 52.38%, and the information entropy is increased by 0.10%, indicating that the encryption effect of the AT-ResNet-CM model is better. It can be seen that the introduction of the attention mechanism effectively improves the encryption performance of the ResNet-CM model and makes it more superior in medical image encryption.

The analysis shows that the attention mechanism can make the model focus more on the region of interest of the medical image. By assigning different weights to the features, a small amount of important information is selected from a large amount of information, thereby effectively improving the encryption quality of the medical image. In addition, the attention mechanism focuses on important information and ignores irrelevant information, which greatly improves the running speed of the model. Therefore, compared with the ResNet-CM model, the AT-ResNet-CM model has higher robustness, security, and efficiency.

The limitation of this method is that the medical image encryption algorithm we designed only uses a simple one-dimensional logistic chaotic mapping method and does not use a more complex and advanced chaotic system for medical image encryption. The confidentiality of this low-dimensional chaotic system is limited, which will restrict the medical image encryption performance to a certain extent. In addition, the generalization of the model is also an aspect that we need to focus on.

## 6. Conclusions

In this paper, we propose a chaotic medical image encryption method using attention mechanism fusion ResNet model (AT-ResNet-CM). This method combines ResNet model and chaotic system to construct a chaotic medical image encryption model ResNet-CM. The high-dimensional feature expression of the medical image is extracted by the residual structure of the ResNet model, and the medical image is encrypted by the pseudo randomness and unpredictability of the chaotic system, which realizes the organic fusion of deep learning and chaotic system. Secondly, the attention mechanism is introduced to improve the ResNet model. The constructed AT-ResNet-CM model can focus more on the region of interest of the medical image and improve the running speed of the model, so as to achieve higher security and timeliness in medical image encryption.

Many comparative experiments were carried out on the AMRG Cardiac MRI Atlas and COVID-19 Chest X-ray datasets. The experimental results show that different deep learning models have achieved good application results in medical image encryption, that is, they all have small horizontal correlation coefficient and large information entropy. The horizontal correlation coefficient of ResNet-CM model is 0.0021, and the information entropy is 7.9887. Compared with the BP, CNN, ResNet and other models used by other authors, our proposed method achieves smaller horizontal correlation coefficient and larger information entropy, indicating that our method performs better in medical image encryption. In addition, the introduction of the attention mechanism further optimizes the encryption performance of the model. The horizontal correlation coefficient of the AT-ResNet-CM model is 0.0010, and the information entropy is 7.9965, which not only improves the two metrics to varying degrees, but also greatly improves the running speed of the model. It can be seen that the medical image encryption method in this paper has higher security, robustness and timeliness, and has higher application prospects in the actual image encryption task. In the future, we consider combining deep learning methods with more complex chaotic systems to solve medical image encryption problems, and also consider improving the generalization of the model so that it has better application effects for various types of medical images to better protect patients' privacy and health information.

## Data availability statement

The original contributions presented in the study are included in the article/supplementary material, further inquiries can be directed to the corresponding author.

## Author contributions

XL and HP: conceptualization, methodology, validation, writing—original draft preparation, writing—review and editing, and visualization. XL: project administration and supervision. All authors have read and agreed to the published version of the manuscript.
